# Femoral revision in total hip arthroplasty using a cementless modular stem: clinical and radiological results with a 8-year follow-up

**DOI:** 10.1007/s00402-023-05066-8

**Published:** 2023-10-24

**Authors:** David Spranz, David Skrobek, Jannis Randoll, Stefan Kinkel, Christian Merle, Tilman Walker, Tobias Renkawitz, Tobias Reiner

**Affiliations:** 1 Department of Orthopaedics, Heidelberg University Hospital, Schlierbacher Landstraße 200 a, 69118 Heidelberg, Germany; 2 Orthopaedicum Darmstadt, Rheinstraße 19, 64283 Darmstadt, Germany; 3 ARCUS Sportklinik Pforzheim, Rastatterstraße 17-19, 75179 Pforzheim, Germany; 4 Department of Orthopedic and Trauma Surgery, Paulinenhilfe, Diakonieklinikum Stuttgart, Rosenbergstraße 38, 70192 Stuttgart, Germany

**Keywords:** Modularity, Hip revision arthroplasty, Implant fracture, Taper damage, MRP-Titan, Periprosthetic femoral fractures, Aseptic loosening

## Abstract

**Introduction:**

Modular femoral components allow for patient-specific restoration of hip joint geometry and the reconstruction of extensive bone defects in revision total hip arthroplasty (THA); however, potential problems of modular implants such as taper corrosion and the risk of implant fracture continue to be of concern. The aim of the present study was to evaluate the clinical and radiological results of a cementless modular revision stem following revision surgery due to aseptic loosening and periprosthetic fracture and to assess patient-reported outcome measures (PROMs) in these patients at mid-term follow-up.

**Materials and methods:**

In this study, a consecutive cohort of 75 patients who underwent primary revision THA at our institution using a modular cementless stem design (MRP-TITAN stem) was retrospectively evaluated at a mean follow-up of 7.7 years. Kaplan–Meier survivorship analyses were performed with revision of the femoral component for any reason as the end point. The Harris-Hip Score, the UCLA Activity Score, the Forgotten Joint Score and the SF-12 Score were used for clinical assessment. We used the Wilcoxon signed rank test to compare pre- and postoperative clinical scores.

**Results:**

Overall stem survival with the endpoint stem re-revision for any reason was 85.4% at a mean follow-up of 7.7 years (range 2.4–14 years). Stem survival was 89.5% in the aseptic loosening group and 78.3% in the periprosthetic fracture group with no statistically significant difference between both groups (*p* = 0.107). One patient had to be revised due to taper fracture. PROMs improved significantly up to the latest follow-up, and radiographic evaluation showed full osseointegration of all stems in this cohort.

**Conclusions:**

Revision THA using a modular cementless titanium revision stem demonstrated adequate clinical and radiological results at mid- to long-term follow-up in this cohort. Cementless revision stems are a useful treatment option to restore the anatomy, especially in deformed hips and in complex revision hip arthroplasty. However, there are some significant disadvantages related to an increased risk of mechanical failure such as corrosion/fretting damage and implant fracture. Future high-quality prospective studies with longer follow-up are necessary to confirm the supposed advantages.

## Introduction

Total hip arthroplasty (THA) has proved to be an excellent and reliable treatment option for end-stage osteoarthritis of the hip with excellent long-term results [1]. The incidence of patients undergoing primary THA has been constantly rising in the past decades, and this tendency is expected to continue in the future [2, 3]. The increase in primary THA results in a higher burden of hip revision surgery. Multiple studies have evaluated epidemiological trends of failed modern hip replacements, and aseptic loosening and periprosthetic femoral fractures are still among the most common reasons for THA revision surgery in the long term [4].

Various theories have been presented to explain the cause of aseptic loosening based on observational, experimental and clinical studies [5]. One of the main mechanisms seems to be the excess production of wear particles, triggering a pro-inflammatory reaction with increased osteoclast differentiation and macrophage activation which can lead to periprosthetic osteolysis and implant failure [1]. Periprosthetic femoral fracture is another clinical important complication after primary THA. These fractures are associated with poor clinical outcome, prolongated functional recovery and a high mortality rate [6]. Among the risk factors for periprosthetic femoral fractures are advanced age, osteoporosis, rheumatoid arthritis and the use of cementless stems in elderly patients [6, 7].

Different cementless modular and non-modular stem designs are available for femoral reconstruction in revision THA. Modular revision stems in THA allow to individually reconstruct hip joint anatomy compromised by bone loss and soft-tissue defects, in order to restore limb length, femoral offset and hip joint stability [8]. Cementless femoral revision stems have become increasingly popular because they provide the potential of long-term biologic implant fixation [9]. Revision THA with conical fluted titanium stems was originally described by Wagner in the 1980s [10, 11]. Axial stability of the implant is achieved by driving the tapered stem into the femoral diaphysis that has been prepared using conical reamers. Longitudinal spines provide rotational stability. Originally being a monoblock design, the subsequent addition of stem modularity improved the versatility of the implant and its broader application in complex revision hip arthroplasty [12–14].

However, mechanical complications associated with stem modularity such as taper fracture and tribocorrosion continue to be of concern [15–17]. There is a relative lack of long-term follow-up studies investigating the clinical and radiological outcome of cementless modular hip revision systems [18]. Furthermore, data on patient-reported outcome measures (PROMs) in patients following hip revision surgery are scarce [19]. Therefore, the aim of the present study was to assess the clinical and radiological results of a modular hip revision system for the treatment of aseptic femoral loosening and periprosthetic femoral fracture and to assess patient-reported outcome measures (PROMs) in these patients at mid- to long-term follow-up.

## Materials and methods

### Patient selection and study cohort

In this single-center study, we retrospectively reviewed data from a consecutive cohort of 75 patients who underwent femoral revision arthroplasty using the cementless MRP-TITAN hip revision system (Peter Brehm GmbH, Weisendorf, Germany) at our institution. Inclusion criteria were patients with primary femoral revision surgery due to aseptic loosening (group AL, 51 patients, 68%) or periprosthetic fracture (group PF, 24 patients, 32%) using a cementless modular femoral revision stem with a minimum follow-up of 24 months. In the AL group, bone defects were classified according to the AAOS classification system of femoral bone deficiencies for revision hip arthroplasty, which was first proposed by D'Antonio et al. and later adopted by the American Academy of Orthopedic Surgeons (AAOS) [20]. The AAOS system classifies defects into segmental (loss of supporting cortical bone) and cavitary (loss of cancellous medullary bone) deficiencies and divides them into six different types (T1–6). Type I (segmental defects) describes a loss of bone of the supporting shell of the femur. Type II defects (cavitary defect) comprise a loss of endosteal bone with an intact cortical shell. Type III (combined defects) is a combination of a type I (segmental defect) and type II (cavitary defect) deficiency. Type IV defects (malalignment) are defined as a loss of the normal femoral geometry due to prior surgery, trauma or disease. Type V deficiencies (stenosis) describe an obliteration of the femoral canal due to trauma, previous fixation devices or bone hypertrophy, and type VI defects (femoral discontinuity) are characterized by the loss of femoral integrity as a consequence of fracture or non-union [20]. The letter H further describes the localization of the defect (H1 defects are located above the inferior border of the lesser trochanter, H2 defects are located within the first 10 cm below the lesser trochanter, and H3 defects are located below the first 10 cm distally to the lesser trochanter). Periprosthetic femoral fractures were classified using the Vancouver classification system introduced by Duncan and Masri [21, 22]. It is currently the most widely used classification system for periprosthetic femoral fractures. The classification includes the anatomical location of the fracture in relation to the stem, the fixation status of the stem and the quality of the remaining bone stock [23]. The national research committee approved the study (S-454/2014), and written informed consent was obtained from all patients before study enrolment.

### Implant design

The modular MRP-TITAN stem (Peter Brehm GmbH, Weisendorf, Germany) was used in all patients. The component is made of titanium alloy (Ti6Al7Nb) and is designed for cementless diaphyseal press-fit fixation [24, 25]. Essentially, the modularity of the implant consists of (1) the distally tapered femoral stem with longitudinal parabolic ribs (available lengths of 80, 140, 200, 260, 320 mm); (2) an optional extension sleeve and (3) three different neck models with a standard 12/14-mm taper. The neck components are available with different neck-stem angles of 130° (37-mm offset) and 123° (47-mm offset). All components are locked in situ with a special proximal expansion bolt [26].

### Clinical and radiographic follow-up

Clinical assessment was performed using the Harris-Hip Score (HHS), the UCLA Activity Score (UCLA), the Forgotten Joint Score (FJS) and the SF-12 Score [27]. Two summary scores are reported from the SF-12 score—a mental component score (MCS-12) and a physical component score (PCS-12). Stem revision was defined as any replacement of one or more of the three components of the MRP-Titan stem (the distal femoral stem and/or the extension sleeve and/or the neck component). Reoperation was defined as any operation without replacement of one of the before-mentioned components. Standard anteroposterior and lateral radiographs of the hip were evaluated with regard to radiolucencies, osteolysis and implant migration up to the latest follow-up. The radiographs were assessed by two independent orthopedic surgeons specialized in THA (D.S. and T.R.). Radiolucencies and osteolysis were evaluated according to the zones established by Gruen et al. [28]. Axial implant migration (subsidence) was measured using fixed landmarks of the prosthesis (such as the modular junction) and fixed anatomical landmarks such as the inter-teardrop line. Implants showing progressive axial migration of more than 5 mm [29], progressive signs of osteolysis or complete periprosthetic radiolucency were classified as loosened. Periarticular ossification was evaluated using the criteria described by Brooker et al. [30].

### Statistical analysis

Exploratory data analysis was used to describe demographic data as mean values with ranges and standard deviations (SD). Continuous data were checked for normal distribution and equal variances. When categorial non-dichotomous variables were to be assessed, Mann–Whitney’s *U* test was used. We used the Wilcoxon signed rank test to compare pre- and postoperative clinical scores and to compare the score values between the AL group and the PF group. Kaplan–Meier survivorship analyses were performed with revision of the femoral component for any reason as the end point. Log-rank test was used to differentiate the survival rates between groups. *p* Values < 0.05 were considered as statistically significant. SPSS^®^ version 26.0 (IBM SPSS Statistics, IBM, Armonk, NY, USA) was used to record and analyze all data.

## Results

### Patient cohort

**Fig. 1:**
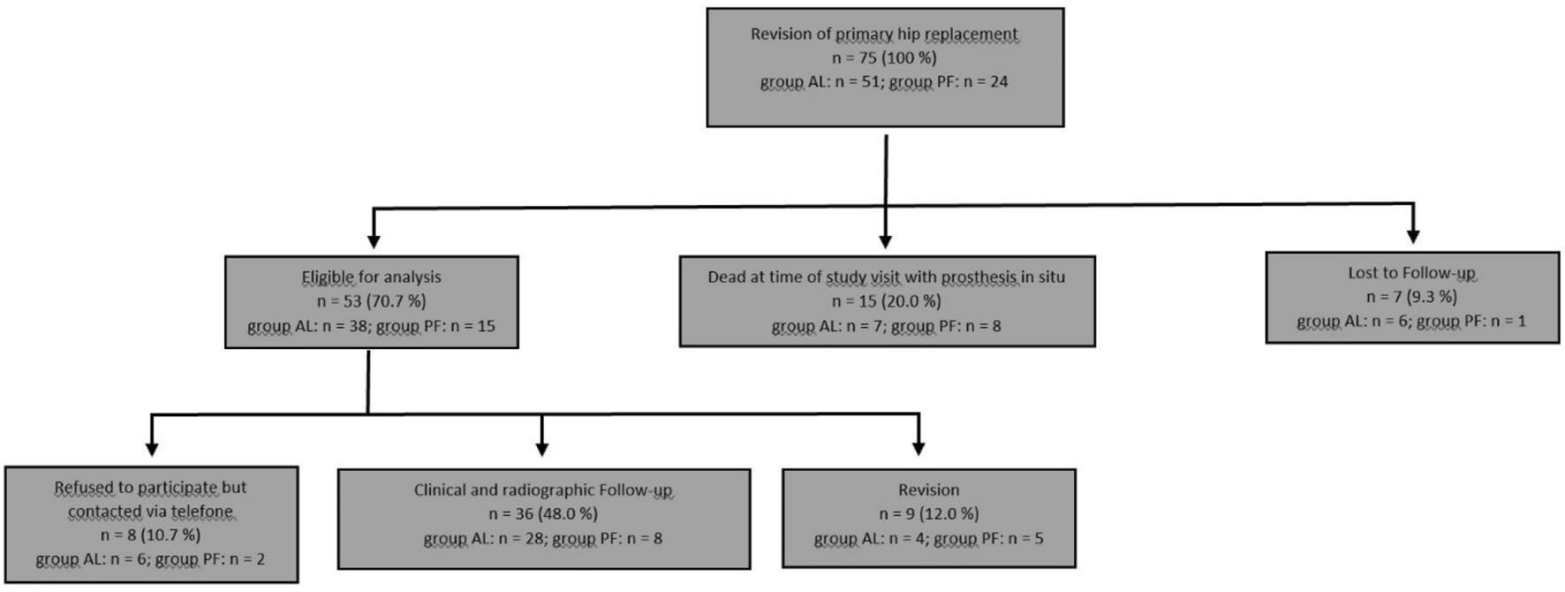
Clinical trial profile and patient flowchart

Figure 1 summarizes the clinical follow-up and patient flowchart.

Of the original cohort (*n* = 75), seven patients (9.3%) were lost to FU (address unknown/foreign country: six patients; death without any information about the stem: 1 patient). Fifteen patients (20.0%) had died from unrelated causes, none of whom had a stem revision at the time of death. From the remaining 53 patients, nine patients (12.0%) underwent stem revision and eight patients (10.7%) refused to participate in the study. All of these patients reported absence of a previous revision surgery. Complete clinical and radiological follow-up data were available in 36 patients at a mean follow-up of 7.7 years (SD 2.7; range 2.4–14.0 years). Table 1 shows patients’ characteristics and demographic data of the study cohort.

**Table 1:** Patient’s characteristics of the cohort with clinical and radiographic FU

Parameter	Overall	Aseptic loosening	Periprosthetic fracture
*n*	36	28	8
Gender (f/m)	20/16	15/13	5/3
Age [mean, standard deviation (SD); range (*r*)]	65.9 (SD 10.1; *r* 45–84)	64.6 (SD 10.6; *r* 45–82)	70.4 (SD 7.2; *r* 63–84)
BMI [mean, standard deviation (SD); range (*r*)]	27.3 (SD 5.5; *r* 20.3–43.6)	27.2 (SD 4.8; *r* 20.3–38.5)	27.8 (SD 8.0; *r* 20.3–43.6)
ASA classification			
1	3	3	0
2	16	11	5
3	17	14	3
Mean latest FU [standard deviation (SD); range (*r*)]	92.0 months (SD 32.5; *r* 29–168)	94.7 months (SD 35.7; *r* 29–168)	82.1 months (SD 15.8; *r* 61–105)
	7.7 years (SD 2.7; *r* 2.4–14.0)	7.9 years (SD 3.0; *r* 2.4–14.0)	6.8 years (SD 1.2; 5.1–8.8)
Vancouver class			
AL			1 (12.5%)
B2			5 (62.5%)
B3			1 (12.5%)
C			1 (12.5%)
AAOS class			
T2H1		2 (7.1%)	
T2H2		7 (25.0%)	
T2H3		8 (28.6%)	
T3H2		6 (21.4%)	
T3H3		4 (14.3%)	
T4H3		1 (3.6%)	

### Survival analysis

**Fig. 2:**
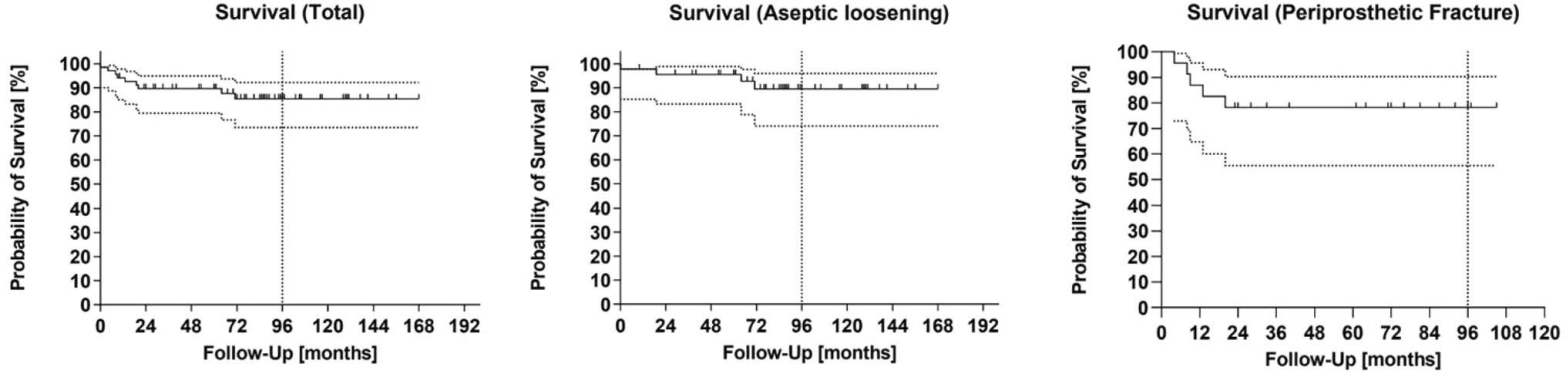
Cumulative survival with the endpoint stem revision for any reason

In summary, the cumulative survival rate at 8 years with the endpoint stem revision for any reason was 85.4% (95% confidence interval 73.5–92.2). At the most recent follow-up, nine patients of the study cohort have had a revision surgery of the stem. Four patients of the group AL (*n* = 45) and five patients of the group PF (*n* = 23) underwent stem revision. In group AL, stem survival was 89.5% at 8 years (95% confidence interval 74.0–96.0). In group PF, stem survival was 78.3% at 8 years (95% confidence interval 55.4–90.3) (see Fig. 2).

There was no statistically significant difference regarding implant survival between both groups (*p* = 0.107). The reasons for revision surgery were deep infection (*n* = 5), periprosthetic femoral fracture (*n* = 1), implant fracture (*n* = 1), progressive subsidence (*n* = 1) and luxation (*n* = 1).

Five patients (6.7%) had a reoperation without revision of the stem. The reasons for reoperation were deep infection (*n* = 1) and aseptic loosening of the cup (*n* = 3). Another patient suffered a periprosthetic fracture without stem loosening (Vancouver type B1) after 4 years. An angle-stable plate osteosynthesis in combination with wire cerclages without stem revision was performed due to a fully osseointegrated stem.

Five different surgeons performed all the operations. All surgeons were certified senior physicians.

### Patient-reported outcome measures

A preoperative HHS was only available in the AL group. Clinical examination showed a statistically significant improvement in the HHS in group AL from 42.7 (SD 21.0, range 9–89) points preoperatively to 73.2 (SD 19.3, range 27–96) points postoperatively (*p* < 0.001). The HHS in group PF was 70.13 (SD 21.52, range 38–100) points postoperatively. There was no significant difference in HHS postoperatively between the two groups (*p* = 0.695). Mean postoperative FJS was 57.81 (SD 32.39, range 4.20–100.00) points in group AL and 50.88 (SD 23.94, range 11.4–91.7) points in group PF. There was no significant difference between the two groups (*p* = 0.588). Median postoperative UCLA was 5.00 points (SD 1.79, range 2–9) in group AL and 4.00 points (SD 2.2, range 2–9) in group PF. There was no significant difference between the two groups (*p* = 0.358).

Mean postoperative PCS-12 was 39.63 (SD 10.48, range 18.78–55.19) points in group AL and 36.76 (SD 12.63, range 18.43–56.68) points in group PF. There was no significant difference between the two groups (*p* = 0.588). Mean postoperative MCS-12 was 52.47 (SD 9.00, range 33.25–68.49) points in group AL and 51.89 (SD 7.19, range 44.55–62.90) points in group PF. There was no significant difference between the two groups (*p* = 0.808). The results of clinical evaluation and postoperative patient-reported outcome measures are summarized in Table 2.

**Table 2:** Postoperative patient-reported outcome measures (PROMs), comparison of the group AL vs. group PF

PROMs	Aseptic loosening	Periprosthetic fracture	*p*-Value
	*n* = 28	*n* = 8	
Harris-hip score	73.2 (SD 19.3)	70.13 (SD 21.52)	*p* = 0.695
Forgotten joint score	57.81 (SD 32.39)	50.88 (SD 23.94)	*p* = 0.588
UCLA activity scale	5.0 (SD 1.79)	4.0 (SD 2.2)	*p* = 0.358
SF-12 physical health	39.6 (SD 10.3)	36.8 (SD 11.8)	*p* = 0.588
SF-12 mental health	52.5 (SD 8.8)	51.9 (SD 6.7)	*p* = 0.808

### Radiographic evaluation

Radiographic evaluation showed full osseointegration of the stem in all cases at latest follow-up with no signs of implant loosening. Periprosthetic radiolucencies were demonstrated in nine cases (25%) that were predominantly located in the proximal Gruen zones (zone 1, 2, 6 and 7). Axial implant migration of < 2 mm was seen in two cases (6%), and initial migration of 5 mm was seen in one case (3%). Migration occurred during the first 6 weeks after surgery in all cases with no further implant migration until the latest FU representing initial settling of the stem. At most recent follow-up, all periprosthetic fractures had achieved radiographic union. Periarticular ossifications were documented in 16 cases (44%) (Brooker 1 *n* = 6, Brooker 2 *n* = 5, Brooker 3 *n* = 5).

## Discussion

Modular stem designs are versatile and offer the opportunity to restore patient’s individual hip joint geometry and the possibility to reconstruct extensive bone defects in complex femoral revision surgery [31]. On the downside, modular taper junctions are susceptible to fretting corrosion and fatigue damage which might lead to metal wear and implant failure in the long term. According to the Australian Orthopaedic Association National Joint Replacement Registry of 2019, femoral stems with modular necks have almost twice the rate of revision compared to fixed neck stems [32]. The aim of the current study was to investigate the clinical and radiological mid- to long-term results of a cementless modular titanium stem in revision THA due to aseptic loosening and periprosthetic femoral fracture.

**Fig. 3:**
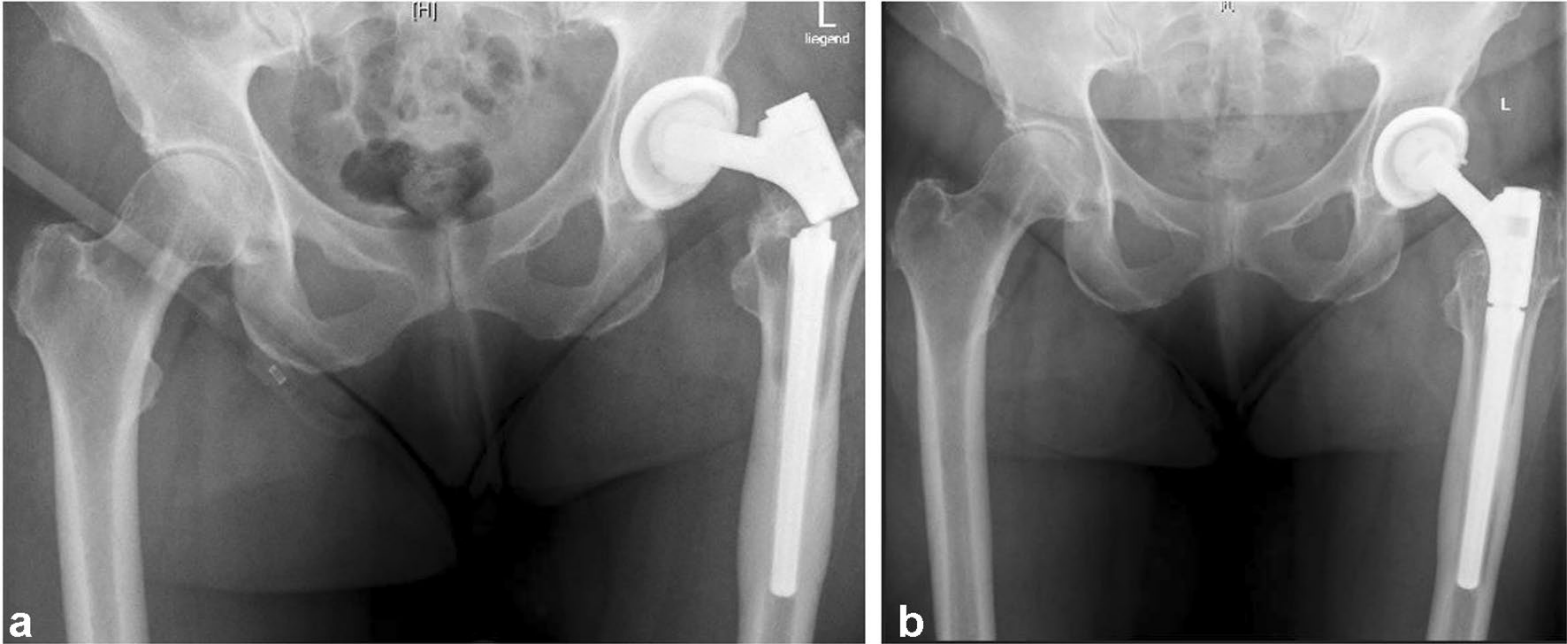
Pelvic radiograph demonstrating implant fracture at the junction between the neck component and the stem 5.3 years after implantation (left side). A 16 × 140 mm stem with a short-length neck segment and a lateralized offset was used in this patient. Notice the extensive osteolysis around the stem in the proximal Gruen zones. Femoral revision was performed with the use of a 17 × 140 mm MRP-Titan stem and a medium-size neck segment with lateralized offset. Follow-up radiograph 7 years after stem re-revision shows full osteointegration of the stem and complete reossification of the osteolysis (right side)

The findings of our study demonstrated good clinical results for the modular cementless revision stem with an overall implant survival of 85% after 8 years. Implant survival seems lower than standard non-modular femoral revision stems; however, since modular stems are mainly used in deformed hips and in complex revision hip arthroplasty, a direct comparison between different patient cohorts has various limitations [33, 34]. One of the major concerns with modular stem designs is fretting corrosion and fatigue damage at the modular junctions that can ultimately lead to material fracture at the femoral component as described by Konan et al. [35]. The incidence of this complication is relevant and the risk increases in patients with a high BMI, a high level of activity, a small medullary canal and in those with severe bone loss in the proximal Gruen zones, which results in a predominantly diaphyseal implant fixation [35]. Garbuz et al. [13] showed one-stem fracture at the modular junction of 31 femoral revisions with a modular distal-fixation fluted tapered stem. This complication was also reported in association with monoblock stem designs intended for distal fixation [36]. Bischel et al. reported four patients with fracture of the taper connection between the stem and the neck after an average of 4.3 years after implantation of the same modular stem system (absolute risk rate of 4.5%; 4 out of 89) [37]. The use of lateralized offset necks in obese patients showed a significantly higher risk of fracture [37]. In contrast, Valtanen et al. [38] reported no modular junction complications in 89 cases with a similar implant at long-term follow-up (> 14 years). In our cohort, one patient (absolute rate 1.3%) required revision due to an implant fracture (see Fig. 3), which also occurred 5.3 years after implantation at the modular junction between the neck and the stem.

This patient met the above-mentioned risk factors with a BMI of 31 kg/m^2^ and an AAOS classification of T2H2. In this case, a short neck version with a lateralized offset was used. This confirms the findings of other authors that a short-length modular neck segment with lateralized offset should be chosen with caution in obese patients due to the biomechanical forces acting at the modular junction [39].

It is well known that revision THA is associated with a significantly poorer functional outcome compared to primary THA [40]. However, only few studies have evaluated the clinical outcome of complex femoral revision surgery with modular stems using patient-reported outcome measures (PROM). The findings of our study suggest sufficient clinical long-term results for femoral revision THA using a modular cementless titanium stem. Previous studies have shown significant differences in reported outcome measures subject to the indication for revision. Turnbull et al. assessed 132 revision THAs (*n* = 59 AL, *n* = 9 PF) at a mean follow-up of 7.9 years postoperatively. They reported significantly better results of mean postoperative OHS and UCLA activity scores in patients who underwent revision THA for aseptic loosening (OHS 23.9 and UCLA activity score 6.0) as compared to those who were revised for periprosthetic fracture or dislocation (OHS 18.0 and UCLA activity score 5.5) [41]. In our study, mean results of HHS, UCLA Activity Score, FJS and the SF-12 Score were also higher in the AL group compared to the PF group, but no statistically significant difference was seen between both groups.

Harada et al. [40] assessed postoperative PROM (UCLA, PCS-12, MCS-12, RCS-12, satisfaction and OHS) in 46 patients who underwent revision THA due to aseptic loosening. Mean postoperative UCLA score in this cohort was 4.5 ± 1.5, mean PCS-12 was 46.2 ± 12.2, and mean MCS-12 was 55.3 ± 9.8 points. These results are comparable to our results. Abdel et al. [42] reported a mean postoperative HHS of 83 points after revision THA using a modular fluted, tapered stem in 44 patients at a mean FU of 4.5 years. Follow-up duration and mean postoperative HHS were comparable to our results.

The survival rate following revision THA seems to be dependent on the indication for the revision. Valtanen et al. [38] demonstrated a survival rate of 85.8% following revision THA using a modular, cementless femoral stem at long-term follow-up (> 14 years). The indications in this study for femoral revision were aseptic loosening, infection and periprosthetic fracture. In particular, THA revision due to periprosthetic fracture seems to be associated with a lower survival rate [43] and a higher frequency of postoperative complications compared to THA revisions due to aseptic loosening [44, 45]. Cnudde et al. [43] investigated the relative survival of patients undergoing revision surgery following elective THA in an observational cohort study. The authors reported a significantly lower relative survival rate following revision due to periprosthetic fracture (0.56) compared to aseptic loosening (0.96) at 10-year follow-up. In our study the survival rate at 8 years following revision THA due to periprosthetic fracture was also lower compared to the survival rate due to aseptic loosening (78.3% vs. 89.5%), but the difference was not statistically significant between these two groups.

The goals of treating periprosthetic fractures include fracture healing and a stable long-term implant fixation [46]. Abdel et al. noted a union rate of 98% (43 of 44 fractures) by using a modular fluted, tapered stem for Vancouver B2 and B3 periprosthetic fractures (follow-up 4.5 years). At a mean follow-up of 4.8 years, Park et al. [47] reported a 92.6% union rate in 27 Vancouver B2 and B3 periprosthetic femoral fractures treated with a modular fluted, tapered stem. Similarly, Mulay et al. noted 91% union rate [48]. Our results confirm that modular fluted, tapered stems provide a high rate of fracture union and implant stability for periprosthetic fractures.

There are some limitations to this study that have to be acknowledged. First, the study is limited by its retrospective design and by the number of patients that could be included in the present study. This was mainly attributed to the fact that femoral revision surgery with the necessity of using a cementless modular stem overall is a relatively rare indication at our institution. In addition, 33% of the patients with periprosthetic fracture were already deceased at the time of follow-up, which reflects the severity and high mortality of this injury. Nevertheless, the small sample size of eight patients, who were available in the PF group at the last follow-up for clinical and radiological assessment, limits the statistical power to detect significant differences regarding patient-reported outcome scores between the two groups. Secondly, the study was limited by the follow-up duration with a mean FU of 8 years. A longer follow-up would be helpful to investigate the long-term survival of cementless modular revision stems, especially because implant fracture due to mechanical failure and fatigue damage might occur at a later point of time. Therefore, additional follow-up studies with longer follow-up durations into the second decade would be helpful to confirm the results of our study and to further evaluate the potential risks and benefits of stem modularity.

In summary, revision total hip arthroplasty with cementless diaphyseal fixation using a modular cementless revision stem demonstrated adequate clinical results and expected survival rates at mid- to long-term follow-up in this cohort. According to the results of our study, a titanium revision stem is a useful treatment option to restore the anatomy in complex revision hip arthroplasty. However, potential problems associated with modular stem designs such as corrosion damage and implant fracture should be further investigated in future studies with longer follow-up duration into the second decade.

## Data Availability

The datasets used and/or analysed during the current study are available from the corresponding author on reasonable request.
